# Supplementary material for: Altered microRNA Transcriptome in Cultured Human Liver Cells upon Infection with Ebola Virus
Source: Int J Mol Sci. 2021 Apr 6;22(7):3792. doi: 10.3390/ijms22073792 (PMC8038836; doi:10.3390/ijms22073792)
Supplement: Supplementary file 1 [file ijms-22-03792-s001.zip › Supplementary_File/C_ GO_Analysis_Results/16-30nt_go_Mayinga-96h-Huh7_vs_Control-96h-Huh7_up.mature_mirna_targets/MF_result(Human).html]

| GO.ID | Term | Ontology | Count | Pop.Hits | List.Total | Pop.Total | Fold.Enrichment | Pvalue | FDR | Enrichment.Score | Gene.Ratio | GENES |
| --- | --- | --- | --- | --- | --- | --- | --- | --- | --- | --- | --- | --- |
| GO:0003713 | transcription coactivator activity | Molecular function | 17 | 317 | 400 | 17548 | 2.35264984227129 | 0.00102264049136082 | 0.476364004581712 | 2.99027701541701 | 0.0425 | NPM1//NCOA1//RARB//RORA//USP16//SUB1//RIPK3//TAF5L//APEX1//NFYC//RAP2C//RNF4//SP4//TAF11//NCOA4//PIAS2//HAND2// |
| GO:0003700 | DNA binding transcription factor activity | Molecular function | 54 | 1551 | 400 | 17548 | 1.52738878143133 | 0.00113248506839421 | 0.476364004581712 | 2.94596751536966 | 0.135 | KLF2//ZNF273//WDHD1//ZHX1//FOXP4//IRX2//DR1//E2F5//CUX2//EBF3//CERS6//ALX3//NR6A1//HOXA9//SMAD5//MITF//NFYC//PAX7//PBX3//PLAGL1//QRICH1//THAP10//ARNTL2//RARB//RFX4//ALX4//RORA//HIF3A//SP4//TRPS1//XBP1//ZNF37A//MIXL1//NCOA1//HAND2//CREB5//FUBP3//GFI1//SUB1//PURB//KAT7//ZNF558//ZNF320//GABPB1//TAF5L//ZNF544//ZNF699//AFF3//REXO4//RNF4//ZNF75D//ZNF329//LZTR1//ZNF251// |
| GO:0016747 | transferase activity, transferring acyl groups other than amino-acyl groups | Molecular function | 13 | 221 | 400 | 17548 | 2.58058823529412 | 0.00173547644538397 | 0.476364004581712 | 2.76058127637988 | 0.0325 | AGPAT5//AGPAT4//MOGAT3//NAT2//CRAT//SATL1//KAT7//TAF5L//NCOA1//TEX30//ZDHHC17//CERS6//LRAT// |
| GO:0140110 | transcription regulator activity | Molecular function | 63 | 1921 | 400 | 17548 | 1.43873503383654 | 0.00190852013201121 | 0.476364004581712 | 2.71930325453843 | 0.1575 | KLF2//ZNF273//WDHD1//ZHX1//FOXP4//IRX2//DR1//E2F5//CUX2//EBF3//CERS6//ALX3//NR6A1//HOXA9//SMAD5//MITF//NFYC//PAX7//PBX3//PLAGL1//QRICH1//THAP10//ARNTL2//RARB//RFX4//ALX4//RORA//HIF3A//SP4//TRPS1//XBP1//ZNF37A//MIXL1//NCOA1//HAND2//CREB5//FUBP3//GFI1//NPM1//SUB1//PURB//KAT7//ZNF558//ZNF320//GABPB1//TAF5L//ZNF544//ZNF699//AFF3//REXO4//RNF4//ZNF75D//ZNF329//LZTR1//ZNF251//SAP30L//USP16//RIPK3//APEX1//RAP2C//TAF11//NCOA4//PIAS2// |
| GO:0016788 | hydrolase activity, acting on ester bonds | Molecular function | 30 | 751 | 400 | 17548 | 1.75246338215712 | 0.00214663433355749 | 0.476364004581712 | 2.66824192875581 | 0.075 | POP4//BCHE//ACPP//PDE1B//CCR5//F2RL2//MTM1//SYNJ1//INPP5B//EXOG//APEX2//APEX1//N4BP2//TSN//PGBD5//ZC3H12A//AEN//CTDSPL2//DUSP16//CDC14A//PTPN7//PTPRG//PTP4A2//PLCXD3//REXO4//DBR1//H6PD//ENOPH1//C2ORF66//RPE65// |
| GO:0046983 | protein dimerization activity | Molecular function | 45 | 1286 | 400 | 17548 | 1.53510886469673 | 0.0026975745166458 | 0.476364004581712 | 2.56902654971415 | 0.1125 | NOD1//CPQ//CANT1//CPOX//EXD1//FKBP1A//CADM2//NR6A1//TIMM9//GRIK2//CD200//NPM1//NPPC//OXCT1//ACPP//BAK1//ABCD3//RPE//CDH24//TFRC//XPNPEP1//SYT16//MIXL1//CD3G//HAND2//ZHX1//DR1//GABPB1//GCLM//TUBB2B//NFYC//H2AFY2//PGLYRP4//ALX4//STX1A//TAF11//XBP1//E2F5//FBXW11//EBF3//SLC24A2//MITF//ARNTL2//HIF3A//NCOA1// |
| GO:0000981 | RNA polymerase II transcription factor activity, sequence-specific DNA binding | Molecular function | 40 | 1108 | 400 | 17548 | 1.58375451263538 | 0.00270322156714388 | 0.476364004581712 | 2.56811835628265 | 0.1 | ARNTL2//PAX7//EBF3//NR6A1//MITF//NFYC//RFX4//MIXL1//FUBP3//HAND2//CUX2//GFI1//SUB1//PBX3//IRX2//PURB//TRPS1//PLAGL1//ALX4//RORA//RARB//KLF2//ZNF273//WDHD1//ZHX1//FOXP4//DR1//E2F5//CERS6//ALX3//HOXA9//SMAD5//QRICH1//THAP10//HIF3A//SP4//XBP1//ZNF37A//NCOA1//CREB5// |
| GO:0042605 | peptide antigen binding | Molecular function | 4 | 28 | 400 | 17548 | 6.26714285714286 | 0.00353078216473294 | 0.476364004581712 | 2.45212907585881 | 0.01 | CLEC4M//DHCR24//CD209//HLA-DPB1// |
| GO:0004519 | endonuclease activity | Molecular function | 9 | 135 | 400 | 17548 | 2.92466666666667 | 0.00381001936578695 | 0.476364004581712 | 2.41907281686164 | 0.0225 | APEX1//TSN//ZC3H12A//POP4//DBR1//APEX2//N4BP2//PGBD5//EXOG// |
| GO:0042578 | phosphoric ester hydrolase activity | Molecular function | 17 | 364 | 400 | 17548 | 2.04887362637363 | 0.00432379670641929 | 0.476364004581712 | 2.36413473362618 | 0.0425 | ACPP//PDE1B//CCR5//F2RL2//MTM1//SYNJ1//INPP5B//APEX2//APEX1//CTDSPL2//DUSP16//CDC14A//PTPN7//PTPRG//PTP4A2//PLCXD3//ENOPH1// |
| GO:0005488 | binding | Molecular function | 360 | 15006 | 400 | 17548 | 1.05245901639344 | 0.0045552389017459 | 0.476364004581712 | 2.34148884130587 | 0.9 | ACBD7//STX1A//PEX5L//MAP3K8//YDJC//PCK1//ENOPH1//EIF4E//NCBP2//DHFR//RARB//RORA//CUX2//PLAGL1//H2AFY2//PURB//ALX4//TRPS1//XBP1//HAND2//EBF3//NR6A1//HOXA9//SMAD5//MITF//NFYC//RFX4//MIXL1//FUBP3//PBX3//NPM1//ZNF704//BCHE//YES1//OSBPL8//GSDMA//RPE65//THBS1//C5AR1//F2R//FSTL3//LRAT//SERPINB3//SERPINB4//TIMP2//PPP1R13B//TRIAP1//DZIP1//WIZ//AEN//ZNF804A//EXOG//KLF2//ZNF273//WDHD1//ZHX1//ZNF488//ZNF597//ZNF558//ZNF320//DR1//E2F5//KDM2A//CERS6//ZNF544//APEX2//APEX1//ZNF699//YBX2//CRLF3//ATRX//QRICH1//THAP10//RAD52//RNF4//HIF3A//TSN//ZNF37A//ZNF75D//CSDE1//ZNF329//SAP30L//ZC3H12A//NCOA1//DDX3Y//PIAS2//ZNF251//CREB5//TRAF4//CKS2//USP51//PRKAA2//KAT7//MCM10//AFF3//SLTM//SUB1//RBM7//POP4//TDRD10//PABPC4L//EXD1//DDX6//EIF1AX//EIF5//MAPKBP1//LARP4B//RPL7L1//NOVA2//NMD3//MRPS7//DBR1//COA1//PSMA1//REXO4//NUFIP2//UPF3A//SNRPF//BTF3//TBCA//TFRC//TNS1//UBE2I//YWHAG//PRR3//MEX3B//EIF3A//SYNJ1//SRSF11//QKI//GTPBP1//G3BP2//CCR5//NF2//SNTG1//TRPC5//EREG//IL2//BTN2A2//BTN2A1//CRAT//CADM2//LTA//CD200//NPPC//DLL4//SMO//CMTM7//CD40LG//FKBP1A//EDIL3//ADAM23//SNAP47//CYP19A1//OGFOD1//SCD//LACC1//ATP7A//CANT1//DGKA//SLC24A2//LTBP1//ASPH//MYL1//FAM20C//RCN2//NECAB3//NECAB1//CDH24//TCHH//VSNL1//PRRG3//SYT16//NAT2//ARPC5//SPEG//CLEC4M//PCGF3//NOD1//IFI30//BTG3//RIPK3//FOXP4//NUS1//CLTA//FSD2//TMEM170A//TRIM71//TPRA1//COX4I1//TMEM199//SPC24//MOB3C//EXOC8//F2RL2//SAMD9L//FGF14//COPG1//ALDOB//KIF1B//FBXO28//OTUD3//FBXW11//CRTC1//ZDHHC17//PIP5K1C//LRRC8B//TSPAN12//HEPACAM2//GABPB1//TCTEX1D2//SENP3//TIMM9//GFI1//TAF5L//OR2L13//COX18//KRT6C//GPX3//CD274//CD209//RFESD//TUBB2B//FAS//INPP5B//KCNQ3//KCNS1//KPNA1//C16ORF87//LMAN1//SNX30//MTM1//TAOK3//PDE1B//UFM1//CDK12//PMP22//GRAMD1C//COMMD8//ACPP//LRRC20//TMEM100//UBE2W//RFK//AGPAT5//N4BP2//NGLY1//NRIP3//B2M//AGPAT4//SMCO4//PSMC6//WDR48//PTPN7//BAK1//PTPRG//ABCD3//RPE//MCCC2//COPS7B//SP4//TACC1//TAF11//DYNLT1//PRDX2//TRPC3//TUFT1//VDAC1//SLC30A2//DNALI1//LAPTM5//KCTD15//MUL1//MOB3B//CALCR//PYROXD1//CCDC68//PTP4A2//CDK2AP1//CLTCL1//KRTAP4-12//RASSF4//PIP5K1A//DCUN1D5//STK24//CUL2//PYROXD2//CDC14A//CCNC//COL23A1//MOB1B//FAM114A1//SYCE1//CEP135//RASSF2//MFAP3L//CEP350//CAMK1G//DYNC1LI2//CMPK1//ETNK2//CDKL1//ARL5B//ARL15//RAP2C//RAB6C//RAB36//PIRT//CCSAP//MAP9//USP16//PGLYRP4//SLC30A5//ZNF185//CHURC1//SYP//TMEM181//CTTNBP2//RUNDC1//SATL1//TSPAN5//DHCR24//XPNPEP1//MARC2//CD3G//GRIK2//SHISA6//SHISA9//SEMA3C//GALNT14//RNPC3//CD1D//RNF152//ASB15//RGS7//RUNDC3B//GCLM//HLA-DPB1//GJB2//VWA1//CPQ//CPOX//OXCT1//IRX2//ALX3//PAX7//MOB3A//SUMF2//STEAP2//XPNPEP2//IDI2//CACNA2D4//ARNTL2//ACAD8//H6PD//NCOA4//IGSF22//WDR1//ALDH1B1//HTR1F// |
| GO:0015491 | cation:cation antiporter activity | Molecular function | 4 | 30 | 400 | 17548 | 5.84933333333333 | 0.00455979364217678 | 0.476364004581712 | 2.34105481130307 | 0.01 | SLC24A2//SLC9C2//SLC9A2//SLC41A1// |
| GO:0022821 | potassium ion antiporter activity | Molecular function | 3 | 16 | 400 | 17548 | 8.225625 | 0.00527856784320208 | 0.476364004581712 | 2.27748389226979 | 0.0075 | SLC24A2//SLC9C2//SLC9A2// |
| GO:0008374 | O-acyltransferase activity | Molecular function | 5 | 50 | 400 | 17548 | 4.387 | 0.00547989170263565 | 0.476364004581712 | 2.26122802425514 | 0.0125 | AGPAT5//AGPAT4//MOGAT3//CRAT//LRAT// |
| GO:0016746 | transferase activity, transferring acyl groups | Molecular function | 13 | 253 | 400 | 17548 | 2.25418972332016 | 0.0055134722752513 | 0.476364004581712 | 2.25857480495393 | 0.0325 | AGPAT5//AGPAT4//MOGAT3//NAT2//CRAT//SATL1//KAT7//TAF5L//NCOA1//TEX30//ZDHHC17//CERS6//LRAT// |
| GO:0015298 | solute:cation antiporter activity | Molecular function | 4 | 34 | 400 | 17548 | 5.16117647058824 | 0.00718492675309866 | 0.521136172103833 | 2.14357765493229 | 0.01 | SLC24A2//SLC9C2//SLC9A2//SLC41A1// |
| GO:0005537 | mannose binding | Molecular function | 3 | 18 | 400 | 17548 | 7.31166666666667 | 0.00743671026598785 | 0.521136172103833 | 2.1286191382831 | 0.0075 | CLEC4M//CD209//LMAN1// |
| GO:0019789 | SUMO transferase activity | Molecular function | 3 | 18 | 400 | 17548 | 7.31166666666667 | 0.00743671026598785 | 0.521136172103833 | 2.1286191382831 | 0.0075 | UBE2I//PIAS2//MUL1// |
| GO:0008134 | transcription factor binding | Molecular function | 24 | 620 | 400 | 17548 | 1.6981935483871 | 0.00838769376008601 | 0.521136172103833 | 2.07635743427878 | 0.06 | TRPS1//XBP1//MIXL1//RORA//CRTC1//NCOA1//DR1//PSMC6//NPM1//HAND2//TAF11//RARB//RNF4//NCOA4//PIAS2//APEX1//EIF4E//E2F5//PPP1R13B//GPX3//NFYC//CDK12//PURB//UBE2I// |
| GO:0003676 | nucleic acid binding | Molecular function | 118 | 4252 | 400 | 17548 | 1.21746472248354 | 0.00850156805165216 | 0.521136172103833 | 2.07050096447704 | 0.295 | EIF4E//NCBP2//DHFR//RARB//RORA//CUX2//PLAGL1//H2AFY2//PURB//ALX4//TRPS1//XBP1//HAND2//EBF3//NR6A1//HOXA9//SMAD5//MITF//NFYC//RFX4//MIXL1//FUBP3//PBX3//NPM1//ZNF704//KLF2//ZNF273//WDHD1//ZHX1//ZNF488//ZNF597//ZNF558//ZNF320//DR1//E2F5//KDM2A//CERS6//ZNF544//APEX2//APEX1//ZNF699//YBX2//CRLF3//ATRX//QRICH1//THAP10//RAD52//RNF4//HIF3A//TSN//ZNF37A//ZNF75D//CSDE1//ZNF329//SAP30L//ZC3H12A//NCOA1//DDX3Y//PIAS2//ZNF251//CREB5//TRAF4//KAT7//MCM10//AFF3//SLTM//SUB1//RBM7//POP4//TDRD10//PABPC4L//EXD1//DDX6//EIF1AX//EIF5//MAPKBP1//LARP4B//RPL7L1//NOVA2//NMD3//MRPS7//DBR1//COA1//PSMA1//REXO4//NUFIP2//UPF3A//SNRPF//BTF3//TBCA//TFRC//TNS1//UBE2I//YWHAG//PRR3//MEX3B//EIF3A//SYNJ1//SRSF11//QKI//GTPBP1//G3BP2//RNPC3//TRIM71//FOXP4//IRX2//ALX3//PAX7//SP4//GABPB1//GFI1//CDK12//ARNTL2//DZIP1//WIZ//AEN//ZNF804A//EXOG// |
| GO:0004527 | exonuclease activity | Molecular function | 6 | 78 | 400 | 17548 | 3.37461538461538 | 0.00874005184992638 | 0.521136172103833 | 2.05848599092737 | 0.015 | APEX2//APEX1//ZC3H12A//REXO4//EXOG//AEN// |
| GO:0005515 | protein binding | Molecular function | 286 | 11565 | 400 | 17548 | 1.08489580631215 | 0.00909123668216518 | 0.521136172103833 | 2.04137703562196 | 0.715 | STX1A//TRPS1//XBP1//MIXL1//RORA//YES1//C5AR1//F2R//FSTL3//THBS1//SERPINB3//SERPINB4//TIMP2//PPP1R13B//TRIAP1//CCR5//NF2//SNTG1//TNS1//TRPC5//YWHAG//EREG//IL2//BTN2A2//BTN2A1//CRAT//CADM2//LTA//CD200//NPPC//DLL4//SMO//CMTM7//CD40LG//FKBP1A//TRAF4//EDIL3//ADAM23//SNAP47//FAS//KCNQ3//PDE1B//CAMK1G//CCSAP//KIF1B//MAP9//CEP350//DYNLT1//CEP135//FGF14//ALDOB//CDH24//DR1//E2F5//GPX3//NFYC//NPM1//CDK12//PURB//RARB//RNF4//TAF11//UBE2I//PIAS2//HAND2//CRTC1//NCOA1//PSMC6//CTTNBP2//QKI//RUNDC1//SMAD5//EXOC8//TSPAN5//DHCR24//EIF4E//HOXA9//MCM10//BCHE//PIP5K1A//MOB1B//CKS2//VDAC1//DDX6//SYT16//CD3G//G3BP2//GRIK2//SHISA6//SHISA9//SEMA3C//NMD3//CD1D//EIF3A//BAK1//RNF152//PEX5L//DCUN1D5//ASB15//UBE2W//MUL1//CUL2//RGS7//CLTA//CLTCL1//ATP7A//ATRX//GCLM//SYP//USP16//USP51//SAP30L//NOD1//SUB1//RIPK3//ZDHHC17//GJB2//CRLF3//ACPP//B2M//PTPRG//RAD52//RPE//VWA1//TFRC//TSN//KCTD15//CPQ//CANT1//CPOX//EXD1//NR6A1//TIMM9//OXCT1//ABCD3//XPNPEP1//WDR48//PIRT//EIF5//STK24//DYNC1LI2//DNALI1//TCTEX1D2//ZHX1//GABPB1//TUBB2B//H2AFY2//PGLYRP4//ALX4//FBXW11//EBF3//SLC24A2//MITF//ARNTL2//HIF3A//CLEC4M//TBCA//LTBP1//NCOA4//ARPC5//IGSF22//ZNF185//WDR1//APEX1//LMAN1//WDHD1//CDK2AP1//WIZ//NAT2//RBM7//SPEG//PCGF3//KLF2//IFI30//POP4//ZNF273//BTG3//KAT7//FOXP4//NUS1//ZNF488//FSD2//TMEM170A//TRIM71//TPRA1//MAP3K8//COX4I1//LACC1//TMEM199//SPC24//MOB3C//ZNF320//EIF1AX//F2RL2//SAMD9L//COPG1//DZIP1//NCBP2//KDM2A//MAPKBP1//LARP4B//FBXO28//OTUD3//PIP5K1C//LRRC8B//TSPAN12//HEPACAM2//SENP3//GFI1//TAF5L//ZNF544//OR2L13//COX18//KRT6C//CD274//CD209//RFESD//INPP5B//ZNF699//KCNS1//KPNA1//C16ORF87//SNX30//ASPH//MTM1//TAOK3//UFM1//PMP22//GRAMD1C//QRICH1//COMMD8//LRRC20//TMEM100//RFK//AGPAT5//PRKAA2//N4BP2//NGLY1//NRIP3//PSMA1//AGPAT4//THAP10//SMCO4//FAM20C//NUFIP2//PTPN7//RCN2//RFX4//NECAB3//MCCC2//COPS7B//AEN//UPF3A//SNRPF//SP4//TACC1//BTF3//PRDX2//TRPC3//TUFT1//VSNL1//SLC30A2//LAPTM5//CSDE1//ZNF329//MOB3B//CALCR//PYROXD1//ZC3H12A//CCDC68//PTP4A2//PRR3//KRTAP4-12//RASSF4//PYROXD2//CDC14A//SYNJ1//CCNC//FUBP3//COL23A1//FAM114A1//SRSF11//SYCE1//CREB5//RASSF2//MFAP3L// |
| GO:0016410 | N-acyltransferase activity | Molecular function | 7 | 103 | 400 | 17548 | 2.98145631067961 | 0.00924855860986741 | 0.521136172103833 | 2.03392594688891 | 0.0175 | NAT2//SATL1//KAT7//TAF5L//NCOA1//TEX30//CERS6// |
| GO:0004518 | nuclease activity | Molecular function | 11 | 214 | 400 | 17548 | 2.255 | 0.0102231125189443 | 0.530052881453746 | 1.99041685919101 | 0.0275 | POP4//APEX2//APEX1//N4BP2//TSN//PGBD5//EXOG//ZC3H12A//AEN//REXO4//DBR1// |
| GO:0016407 | acetyltransferase activity | Molecular function | 7 | 105 | 400 | 17548 | 2.92466666666667 | 0.0102247855218701 | 0.530052881453746 | 1.99034579311478 | 0.0175 | NAT2//CRAT//SATL1//KAT7//TAF5L//NCOA1//TEX30// |
| GO:0004521 | endoribonuclease activity | Molecular function | 5 | 60 | 400 | 17548 | 3.65583333333333 | 0.0117494911558679 | 0.581917884968054 | 1.92998094130577 | 0.0125 | APEX1//POP4//DBR1//TSN//ZC3H12A// |
| GO:0003677 | DNA binding | Molecular function | 74 | 2520 | 400 | 17548 | 1.28824603174603 | 0.0121232892701678 | 0.581917884968054 | 1.91637953214373 | 0.185 | RARB//RORA//CUX2//PLAGL1//H2AFY2//PURB//ALX4//TRPS1//XBP1//HAND2//EBF3//NR6A1//HOXA9//SMAD5//MITF//NFYC//RFX4//MIXL1//FUBP3//PBX3//NPM1//ZNF704//APEX1//KAT7//MCM10//AFF3//SLTM//SUB1//RAD52//TSN//UPF3A//KLF2//FOXP4//ZNF558//IRX2//ALX3//PAX7//SP4//ZNF329//GABPB1//GFI1//CDK12//SAP30L//ARNTL2//ZNF273//WDHD1//ZHX1//ZNF488//ZNF597//ZNF320//DR1//E2F5//KDM2A//CERS6//ZNF544//APEX2//ZNF699//YBX2//CRLF3//ATRX//QRICH1//THAP10//RNF4//HIF3A//ZNF37A//ZNF75D//CSDE1//ZC3H12A//NCOA1//DDX3Y//PIAS2//ZNF251//CREB5//TRAF4// |
| GO:0046915 | transition metal ion transmembrane transporter activity | Molecular function | 4 | 40 | 400 | 17548 | 4.387 | 0.0127275210241065 | 0.589102401687215 | 1.89525617689554 | 0.01 | ATP7A//SLC30A5//SLC30A2//TFRC// |
| GO:0008080 | N-acetyltransferase activity | Molecular function | 6 | 86 | 400 | 17548 | 3.0606976744186 | 0.013763056458963 | 0.615066247269519 | 1.86128510855269 | 0.015 | NAT2//SATL1//KAT7//TAF5L//NCOA1//TEX30// |
| GO:0001786 | phosphatidylserine binding | Molecular function | 4 | 42 | 400 | 17548 | 4.17809523809524 | 0.0150458489813388 | 0.649980675993836 | 1.82258330160897 | 0.01 | OSBPL8//GSDMA//RPE65//THBS1// |
| GO:0050681 | androgen receptor binding | Molecular function | 4 | 43 | 400 | 17548 | 4.08093023255814 | 0.0162983310516841 | 0.672448612416939 | 1.78785686505331 | 0.01 | RNF4//NCOA4//NCOA1//PIAS2// |
| GO:0008081 | phosphoric diester hydrolase activity | Molecular function | 6 | 91 | 400 | 17548 | 2.89252747252747 | 0.0177666484529019 | 0.672448612416939 | 1.75039449091682 | 0.015 | PDE1B//CCR5//F2RL2//APEX2//APEX1//PLCXD3// |
| GO:0048029 | monosaccharide binding | Molecular function | 5 | 69 | 400 | 17548 | 3.17898550724638 | 0.0205056839210286 | 0.672448612416939 | 1.68812574121798 | 0.0125 | CLEC4M//CD209//LMAN1//OGFOD1//ALDOB// |
| GO:0008296 | 3'-5'-exodeoxyribonuclease activity | Molecular function | 2 | 10 | 400 | 17548 | 8.774 | 0.0206645780658114 | 0.672448612416939 | 1.68477345781806 | 0.005 | APEX2//APEX1// |
| GO:0015279 | store-operated calcium channel activity | Molecular function | 2 | 10 | 400 | 17548 | 8.774 | 0.0206645780658114 | 0.672448612416939 | 1.68477345781806 | 0.005 | TRPC3//TRPC5// |
| GO:0140078 | class I DNA-(apurinic or apyrimidinic site) endonuclease activity | Molecular function | 2 | 10 | 400 | 17548 | 8.774 | 0.0206645780658114 | 0.672448612416939 | 1.68477345781806 | 0.005 | APEX2//APEX1// |
| GO:0140080 | class III/IV DNA-(apurinic or apyrimidinic site) endonuclease activity | Molecular function | 2 | 10 | 400 | 17548 | 8.774 | 0.0206645780658114 | 0.672448612416939 | 1.68477345781806 | 0.005 | APEX2//APEX1// |
| GO:0072509 | divalent inorganic cation transmembrane transporter activity | Molecular function | 3 | 26 | 400 | 17548 | 5.06192307692308 | 0.0207237460347082 | 0.672448612416939 | 1.68353173854283 | 0.0075 | SLC30A5//SLC30A2//SLC41A1// |
| GO:0034593 | phosphatidylinositol bisphosphate phosphatase activity | Molecular function | 3 | 27 | 400 | 17548 | 4.87444444444444 | 0.0229290823937684 | 0.672448612416939 | 1.63961332509112 | 0.0075 | INPP5B//SYNJ1//MTM1// |
| GO:0015386 | potassium:proton antiporter activity | Molecular function | 2 | 11 | 400 | 17548 | 7.97636363636364 | 0.0248805707195957 | 0.672448612416939 | 1.60413966186191 | 0.005 | SLC9C2//SLC9A2// |
| GO:0043023 | ribosomal large subunit binding | Molecular function | 2 | 11 | 400 | 17548 | 7.97636363636364 | 0.0248805707195957 | 0.672448612416939 | 1.60413966186191 | 0.005 | NPM1//NMD3// |
| GO:0046030 | inositol trisphosphate phosphatase activity | Molecular function | 2 | 11 | 400 | 17548 | 7.97636363636364 | 0.0248805707195957 | 0.672448612416939 | 1.60413966186191 | 0.005 | INPP5B//SYNJ1// |
| GO:0052743 | inositol tetrakisphosphate phosphatase activity | Molecular function | 2 | 11 | 400 | 17548 | 7.97636363636364 | 0.0248805707195957 | 0.672448612416939 | 1.60413966186191 | 0.005 | INPP5B//SYNJ1// |
| GO:0042802 | identical protein binding | Molecular function | 50 | 1661 | 400 | 17548 | 1.32059000602047 | 0.0254642134291764 | 0.672448612416939 | 1.59406973431773 | 0.125 | NOD1//CPQ//CANT1//CPOX//EXD1//FKBP1A//CADM2//NR6A1//TIMM9//GRIK2//CD200//NPM1//NPPC//OXCT1//ACPP//BAK1//ABCD3//RPE//CDH24//TFRC//XPNPEP1//SYT16//MIXL1//CD3G//HAND2//SUB1//RIPK3//ALDOB//ZDHHC17//GJB2//FAS//CRLF3//MCM10//B2M//PSMC6//PTPRG//RAD52//BCHE//RNF4//VWA1//SYP//DYNLT1//THBS1//TSN//VDAC1//XBP1//YWHAG//KCTD15//MUL1//TRAF4// |
| GO:0001618 | virus receptor activity | Molecular function | 5 | 74 | 400 | 17548 | 2.96418918918919 | 0.0268331046579872 | 0.672448612416939 | 1.57132907545232 | 0.0125 | CLEC4M//CCR5//CD209//SERPINB3//TFRC// |
| GO:0104005 | hijacked molecular function | Molecular function | 5 | 74 | 400 | 17548 | 2.96418918918919 | 0.0268331046579872 | 0.672448612416939 | 1.57132907545232 | 0.0125 | CLEC4M//CCR5//CD209//SERPINB3//TFRC// |
| GO:0004725 | protein tyrosine phosphatase activity | Molecular function | 6 | 100 | 400 | 17548 | 2.6322 | 0.0268570292246151 | 0.672448612416939 | 1.57094202825371 | 0.015 | PTP4A2//PTPRG//MTM1//PTPN7//DUSP16//CDC14A// |
| GO:0005251 | delayed rectifier potassium channel activity | Molecular function | 3 | 29 | 400 | 17548 | 4.53827586206897 | 0.0277069307539739 | 0.672448612416939 | 1.55741158069339 | 0.0075 | KCNC4//KCNQ3//KCNS1// |
| GO:0016411 | acylglycerol O-acyltransferase activity | Molecular function | 3 | 29 | 400 | 17548 | 4.53827586206897 | 0.0277069307539739 | 0.672448612416939 | 1.55741158069339 | 0.0075 | AGPAT5//AGPAT4//MOGAT3// |
| GO:0072341 | modified amino acid binding | Molecular function | 5 | 75 | 400 | 17548 | 2.92466666666667 | 0.0282318452526095 | 0.672448612416939 | 1.54926073514263 | 0.0125 | OSBPL8//GSDMA//RPE65//THBS1//DHFR// |
| GO:0003743 | translation initiation factor activity | Molecular function | 4 | 51 | 400 | 17548 | 3.44078431372549 | 0.0286671738941537 | 0.672448612416939 | 1.54261511914529 | 0.01 | EIF1AX//EIF4E//EIF5//EIF3A// |
| GO:0043565 | sequence-specific DNA binding | Molecular function | 36 | 1139 | 400 | 17548 | 1.38658472344162 | 0.0293791733722408 | 0.672448612416939 | 1.53196042791012 | 0.09 | RARB//RORA//CUX2//PLAGL1//H2AFY2//PURB//ALX4//TRPS1//XBP1//HAND2//EBF3//NR6A1//HOXA9//SMAD5//MITF//NFYC//RFX4//MIXL1//FUBP3//PBX3//ZNF704//KAT7//MCM10//APEX1//UPF3A//ARNTL2//KLF2//FOXP4//ZNF558//IRX2//ALX3//PAX7//SP4//TSN//ZNF329//SLTM// |
| GO:0048185 | activin binding | Molecular function | 2 | 12 | 400 | 17548 | 7.31166666666667 | 0.0294129130759012 | 0.672448612416939 | 1.53146196052736 | 0.005 | FSTL3//FKBP1A// |
| GO:0052629 | phosphatidylinositol-3,5-bisphosphate 3-phosphatase activity | Molecular function | 2 | 12 | 400 | 17548 | 7.31166666666667 | 0.0294129130759012 | 0.672448612416939 | 1.53146196052736 | 0.005 | MTM1//SYNJ1// |
| GO:0106019 | phosphatidylinositol-4,5-bisphosphate phosphatase activity | Molecular function | 2 | 12 | 400 | 17548 | 7.31166666666667 | 0.0294129130759012 | 0.672448612416939 | 1.53146196052736 | 0.005 | INPP5B//SYNJ1// |
| GO:0052866 | phosphatidylinositol phosphate phosphatase activity | Molecular function | 3 | 30 | 400 | 17548 | 4.387 | 0.0302788348775069 | 0.672448612416939 | 1.51886084040066 | 0.0075 | MTM1//SYNJ1//INPP5B// |
| GO:0030145 | manganese ion binding | Molecular function | 4 | 52 | 400 | 17548 | 3.37461538461538 | 0.0305162575548455 | 0.672448612416939 | 1.51546872834948 | 0.01 | SLC24A2//PCK1//FAM20C//XPNPEP1// |
| GO:0016787 | hydrolase activity | Molecular function | 74 | 2636 | 400 | 17548 | 1.23155538694992 | 0.0312888125404814 | 0.672448612416939 | 1.50461091869792 | 0.185 | POP4//NGLY1//ATRX//DDX6//DYNLT1//DNALI1//DYNC1LI2//KIF1B//LYZL6//EIF5//TUBB2B//RAP2C//RAB6C//GTPBP1//RAB36//BCHE//ACPP//DDX3Y//ATP7A//PDE1B//PSMA8//SENP3//PSMA1//ST14//XPNPEP1//XPNPEP2//CPQ//NRIP3//USP16//CTSF//ADAM23//GZMK//PRSS22//CANT1//SAP30L//HYAL4//CCR5//F2RL2//MTM1//SYNJ1//INPP5B//EXOG//APEX2//APEX1//N4BP2//TSN//PGBD5//ZC3H12A//AEN//CTDSPL2//DUSP16//CDC14A//PTPN7//PTPRG//PTP4A2//USP51//OTUD3//PLCXD3//SEC11A//REXO4//DBR1//PGLYRP4//VNN1//PSMC6//ABCD3//H6PD//WDR48//ATP6V0E1//ENOPH1//C2ORF66//RPE65//YDJC//IDI2//TEX30// |
| GO:1901363 | heterocyclic compound binding | Molecular function | 158 | 6135 | 400 | 17548 | 1.12982233088835 | 0.031356968827383 | 0.672448612416939 | 1.50366592574418 | 0.395 | EIF4E//NCBP2//DHFR//RARB//RORA//CUX2//PLAGL1//H2AFY2//PURB//ALX4//TRPS1//XBP1//HAND2//EBF3//NR6A1//HOXA9//SMAD5//MITF//NFYC//RFX4//MIXL1//FUBP3//PBX3//NPM1//ZNF704//DZIP1//WIZ//AEN//ZNF804A//EXOG//KLF2//ZNF273//WDHD1//ZHX1//ZNF488//ZNF597//ZNF558//ZNF320//DR1//E2F5//KDM2A//CERS6//ZNF544//APEX2//APEX1//ZNF699//YBX2//CRLF3//ATRX//QRICH1//THAP10//RAD52//RNF4//HIF3A//TSN//ZNF37A//ZNF75D//CSDE1//ZNF329//SAP30L//ZC3H12A//NCOA1//DDX3Y//PIAS2//ZNF251//CREB5//TRAF4//KAT7//MCM10//AFF3//SLTM//SUB1//RBM7//POP4//TDRD10//PABPC4L//EXD1//DDX6//EIF1AX//EIF5//MAPKBP1//LARP4B//RPL7L1//NOVA2//NMD3//MRPS7//DBR1//COA1//PSMA1//REXO4//NUFIP2//UPF3A//SNRPF//BTF3//TBCA//TFRC//TNS1//UBE2I//YWHAG//PRR3//MEX3B//EIF3A//SYNJ1//SRSF11//QKI//GTPBP1//G3BP2//SPEG//NOD1//RIPK3//MAP3K8//DGKA//DYNC1LI2//KIF1B//PIP5K1C//TAOK3//CMPK1//CDK12//ATP7A//ETNK2//UBE2W//RFK//PRKAA2//N4BP2//FAM20C//PSMC6//CAMK1G//ABCD3//MCCC2//YES1//PIP5K1A//STK24//CDKL1//ARL5B//TUBB2B//PCK1//ARL15//RAP2C//RAB6C//RAB36//FKBP1A//CYP19A1//MARC2//RNPC3//TRIM71//FOXP4//IRX2//ALX3//PAX7//SP4//GABPB1//GFI1//DHCR24//ACAD8//H6PD//ALDH1B1//HTR1F//ARNTL2// |
| GO:0097159 | organic cyclic compound binding | Molecular function | 160 | 6221 | 400 | 17548 | 1.12830734608584 | 0.0314482031328761 | 0.672448612416939 | 1.5024041639475 | 0.4 | EIF4E//NCBP2//DHFR//RARB//RORA//CUX2//PLAGL1//H2AFY2//PURB//ALX4//TRPS1//XBP1//HAND2//EBF3//NR6A1//HOXA9//SMAD5//MITF//NFYC//RFX4//MIXL1//FUBP3//PBX3//NPM1//ZNF704//DZIP1//WIZ//AEN//ZNF804A//EXOG//KLF2//ZNF273//WDHD1//ZHX1//ZNF488//ZNF597//ZNF558//ZNF320//DR1//E2F5//KDM2A//CERS6//ZNF544//APEX2//APEX1//ZNF699//YBX2//CRLF3//ATRX//QRICH1//THAP10//RAD52//RNF4//HIF3A//TSN//ZNF37A//ZNF75D//CSDE1//ZNF329//SAP30L//ZC3H12A//NCOA1//DDX3Y//PIAS2//ZNF251//CREB5//TRAF4//KAT7//MCM10//AFF3//SLTM//SUB1//RBM7//POP4//TDRD10//PABPC4L//EXD1//DDX6//EIF1AX//EIF5//MAPKBP1//LARP4B//RPL7L1//NOVA2//NMD3//MRPS7//DBR1//COA1//PSMA1//REXO4//NUFIP2//UPF3A//SNRPF//BTF3//TBCA//TFRC//TNS1//UBE2I//YWHAG//PRR3//MEX3B//EIF3A//SYNJ1//SRSF11//QKI//GTPBP1//G3BP2//SPEG//NOD1//RIPK3//MAP3K8//DGKA//DYNC1LI2//KIF1B//PIP5K1C//TAOK3//CMPK1//CDK12//ATP7A//ETNK2//UBE2W//RFK//PRKAA2//N4BP2//FAM20C//PSMC6//CAMK1G//ABCD3//MCCC2//YES1//PIP5K1A//STK24//CDKL1//ARL5B//TUBB2B//PCK1//ARL15//RAP2C//RAB6C//RAB36//FKBP1A//OSBPL8//SYP//CYP19A1//MARC2//RNPC3//TRIM71//FOXP4//IRX2//ALX3//PAX7//SP4//GABPB1//GFI1//DHCR24//ACAD8//H6PD//ALDH1B1//HTR1F//ARNTL2// |
| GO:0005164 | tumor necrosis factor receptor binding | Molecular function | 3 | 31 | 400 | 17548 | 4.24548387096774 | 0.032971997654916 | 0.672448612416939 | 1.48185473971688 | 0.0075 | LTA//CD40LG//TRAF4// |
| GO:0003906 | DNA-(apurinic or apyrimidinic site) endonuclease activity | Molecular function | 2 | 13 | 400 | 17548 | 6.74923076923077 | 0.0342450682249367 | 0.672448612416939 | 1.46540196422751 | 0.005 | APEX2//APEX1// |
| GO:0015385 | sodium:proton antiporter activity | Molecular function | 2 | 13 | 400 | 17548 | 6.74923076923077 | 0.0342450682249367 | 0.672448612416939 | 1.46540196422751 | 0.005 | SLC9C2//SLC9A2// |
| GO:0016863 | intramolecular oxidoreductase activity, transposing C=C bonds | Molecular function | 2 | 13 | 400 | 17548 | 6.74923076923077 | 0.0342450682249367 | 0.672448612416939 | 1.46540196422751 | 0.005 | IDI2//EBPL// |
| GO:0034595 | phosphatidylinositol phosphate 5-phosphatase activity | Molecular function | 2 | 13 | 400 | 17548 | 6.74923076923077 | 0.0342450682249367 | 0.672448612416939 | 1.46540196422751 | 0.005 | INPP5B//SYNJ1// |
| GO:0070679 | inositol 1,4,5 trisphosphate binding | Molecular function | 2 | 13 | 400 | 17548 | 6.74923076923077 | 0.0342450682249367 | 0.672448612416939 | 1.46540196422751 | 0.005 | TRPC3//TRPC5// |
| GO:0003712 | transcription cofactor activity | Molecular function | 20 | 566 | 400 | 17548 | 1.55017667844523 | 0.0357553689787519 | 0.674549109237392 | 1.44665873588324 | 0.05 | NPM1//NCOA1//USP16//SUB1//RIPK3//TAF5L//APEX1//NFYC//RAP2C//RNF4//SP4//TAF11//NCOA4//PIAS2//HAND2//ZHX1//DR1//RARB//RORA//SAP30L// |
| GO:0016891 | endoribonuclease activity, producing 5'-phosphomonoesters | Molecular function | 3 | 32 | 400 | 17548 | 4.1128125 | 0.0357856493178848 | 0.674549109237392 | 1.44629109825957 | 0.0075 | APEX1//POP4//DBR1// |
| GO:0043178 | alcohol binding | Molecular function | 5 | 80 | 400 | 17548 | 2.741875 | 0.0359134942418056 | 0.674549109237392 | 1.44474233767239 | 0.0125 | OSBPL8//SYP//LRAT//TRPC3//TRPC5// |
| GO:0043169 | cation binding | Molecular function | 112 | 4225 | 400 | 17548 | 1.16294437869822 | 0.0377476432156497 | 0.68935090291389 | 1.42311015848221 | 0.28 | MAP3K8//YDJC//PCK1//ENOPH1//CYP19A1//OGFOD1//SCD//LACC1//ATP7A//EDIL3//CANT1//DGKA//SLC24A2//LTBP1//ASPH//MYL1//DLL4//FAM20C//RCN2//NECAB3//NECAB1//CDH24//THBS1//TCHH//VSNL1//PRRG3//SYT16//MEX3B//USP16//KAT7//TRIM71//USP51//KDM2A//NR6A1//TIMM9//APEX2//PGLYRP4//RARB//RNF4//RORA//SLC30A5//TIMP2//TRPS1//ZNF75D//ZNF185//SAP30L//PIAS2//CHURC1//TRAF4//SATL1//XPNPEP1//MARC2//RPE65//BCHE//CLEC4M//PCGF3//KLF2//CPQ//ZNF273//ZHX1//FOXP4//ZNF488//MOB3A//ZNF597//ZNF558//MOB3C//ZNF320//RNF152//DZIP1//EBF3//SUMF2//STEAP2//GFI1//ZNF544//CD209//RFESD//APEX1//INPP5B//ZNF699//LMAN1//SMAD5//DBR1//PDE1B//PLAGL1//ATRX//RFK//MCM10//PRKAA2//NGLY1//THAP10//BAK1//WIZ//RPE//ZNF704//SP4//XPNPEP2//ZNF37A//MUL1//GALNT14//ZNF329//MOB3B//ZC3H12A//PRR3//STK24//ZNF251//IDI2//ZNF804A//MOB1B//CACNA2D4//CREB5//EXOG//HTR1F// |
| GO:0001205 | transcriptional activator activity, RNA polymerase II distal enhancer sequence-specific DNA binding | Molecular function | 3 | 33 | 400 | 17548 | 3.98818181818182 | 0.0387188549397689 | 0.68935090291389 | 1.41207749487328 | 0.0075 | SUB1//PBX3//MIXL1// |
| GO:0003688 | DNA replication origin binding | Molecular function | 2 | 14 | 400 | 17548 | 6.26714285714286 | 0.0393610855058857 | 0.68935090291389 | 1.40493293303756 | 0.005 | KAT7//MCM10// |
| GO:0005451 | monovalent cation:proton antiporter activity | Molecular function | 2 | 14 | 400 | 17548 | 6.26714285714286 | 0.0393610855058857 | 0.68935090291389 | 1.40493293303756 | 0.005 | SLC9C2//SLC9A2// |
| GO:0045504 | dynein heavy chain binding | Molecular function | 2 | 14 | 400 | 17548 | 6.26714285714286 | 0.0393610855058857 | 0.68935090291389 | 1.40493293303756 | 0.005 | DYNC1LI2//DNALI1// |
| GO:0004402 | histone acetyltransferase activity | Molecular function | 4 | 57 | 400 | 17548 | 3.07859649122807 | 0.0407979054535212 | 0.704987806236846 | 1.38936213282502 | 0.01 | KAT7//TAF5L//NCOA1//TEX30// |
| GO:0004438 | phosphatidylinositol-3-phosphatase activity | Molecular function | 2 | 15 | 400 | 17548 | 5.84933333333333 | 0.0447455824821727 | 0.735422493719031 | 1.34924983405344 | 0.005 | MTM1//SYNJ1// |
| GO:0052744 | phosphatidylinositol monophosphate phosphatase activity | Molecular function | 2 | 15 | 400 | 17548 | 5.84933333333333 | 0.0447455824821727 | 0.735422493719031 | 1.34924983405344 | 0.005 | MTM1//SYNJ1// |
| GO:0106018 | phosphatidylinositol-3,5-bisphosphate phosphatase activity | Molecular function | 2 | 15 | 400 | 17548 | 5.84933333333333 | 0.0447455824821727 | 0.735422493719031 | 1.34924983405344 | 0.005 | SYNJ1//MTM1// |
| GO:0046872 | metal ion binding | Molecular function | 109 | 4131 | 400 | 17548 | 1.15754780924716 | 0.0453777259688992 | 0.735422493719031 | 1.34315727186898 | 0.2725 | MAP3K8//YDJC//PCK1//ENOPH1//CYP19A1//OGFOD1//SCD//LACC1//ATP7A//EDIL3//CANT1//DGKA//SLC24A2//LTBP1//ASPH//MYL1//DLL4//FAM20C//RCN2//NECAB3//NECAB1//CDH24//THBS1//TCHH//VSNL1//PRRG3//SYT16//MEX3B//USP16//KAT7//TRIM71//USP51//KDM2A//NR6A1//TIMM9//APEX2//PGLYRP4//RARB//RNF4//RORA//SLC30A5//TIMP2//TRPS1//ZNF75D//ZNF185//SAP30L//PIAS2//CHURC1//TRAF4//XPNPEP1//MARC2//CLEC4M//PCGF3//KLF2//CPQ//ZNF273//ZHX1//FOXP4//ZNF488//MOB3A//ZNF597//ZNF558//MOB3C//ZNF320//RNF152//DZIP1//EBF3//SUMF2//STEAP2//GFI1//ZNF544//CD209//RFESD//APEX1//INPP5B//ZNF699//LMAN1//SMAD5//DBR1//PDE1B//PLAGL1//ATRX//RFK//MCM10//PRKAA2//NGLY1//THAP10//BAK1//WIZ//RPE//RPE65//ZNF704//SP4//XPNPEP2//ZNF37A//MUL1//GALNT14//ZNF329//MOB3B//ZC3H12A//PRR3//STK24//ZNF251//IDI2//ZNF804A//MOB1B//CACNA2D4//CREB5//EXOG// |
| GO:0061733 | peptide-lysine-N-acetyltransferase activity | Molecular function | 4 | 59 | 400 | 17548 | 2.97423728813559 | 0.0453964502295698 | 0.735422493719031 | 1.34297810542445 | 0.01 | KAT7//TAF5L//NCOA1//TEX30// |
| GO:0016791 | phosphatase activity | Molecular function | 11 | 270 | 400 | 17548 | 1.7872962962963 | 0.0459938753792192 | 0.735902006067507 | 1.33729999584231 | 0.0275 | ACPP//MTM1//SYNJ1//INPP5B//CTDSPL2//DUSP16//CDC14A//PTPN7//PTPRG//PTP4A2//ENOPH1// |
| GO:0032182 | ubiquitin-like protein binding | Molecular function | 5 | 86 | 400 | 17548 | 2.55058139534884 | 0.0466818251216547 | 0.73780055314225 | 1.33085217266162 | 0.0125 | RNF4//USP16//CKS2//WDR48//DCUN1D5// |
